# Interfacial and Hydration‐Associated Lithium Retention During FePO_4_ Regeneration from Spent LiFePO_4_ Cathodes

**DOI:** 10.1002/advs.78485

**Published:** 2026-10-26

**Authors:** Fei Han, Langyi Xiao, Genping Yi, Tian Liu, Shoudong Huang, Haonan Yan, Guang Yang, Liming Yang, Xubiao Luo

**Affiliations:** ^1^ School of Life Sciences Key Laboratory of Jiangxi Province for Functional Biology and Pollution Control in Red Soil Regions Jinggangshan University Ji'an China; ^2^ National‐Local Joint Engineering Research Center of Heavy Metals Pollutants Control and Resource Utilization Nanchang Hangkong University Nanchang China; ^3^ School of Materials Science and Engineering Beihang University Beijing China

**Keywords:** hydrated structural evolution, interfacial association, iron phosphate recovery, lithium retention, spent LiFePO_4_ recycling

## Abstract

Selective FePO_4_ precipitation is used to separate iron and phosphorus from lithium during hydrometallurgical recycling of spent LiFePO_4_, yet measurable lithium can remain in the regenerated FePO_4_ precursor. Here, lithium partitioning was examined in a simulated Fe–P system, a commercial LiFePO_4_‐derived leachate, and a spent black‐mass‐derived leachate. Sequential washing resolved the lithium associated with the precipitates into fractions with different accessibility. Under representative conditions, approximately 70% of the retained lithium was washing‐removable, whereas approximately 30% remained after three washing cycles. Contact with lithium‐free FePO_4_ resulted in an apparent 2.13% decrease in dissolved Li concentration, approaching a plateau within 10 min. Thermodynamic analysis indicated that the formation of the considered discrete lithium‐containing precipitates was unfavorable, while changes in hydration and local Fe–O–P environments correlated with the washing‐retained fraction. These results support a proposed precipitation‐associated model involving lithium retention in environments with different accessibility within hydrated amorphous FePO_4_. The findings identify precipitation and washing as linked control stages for managing lithium carryover during FePO_4_ regeneration.

## Graphical abstract

During iron phosphate regeneration, sequential washing distinguishes lithium fractions by accessibility: approximately 70% of precipitate‐associated lithium is washing‐removable, while approximately 30% remains after three cycles. Comparisons of simulated and practical systems associate retained lithium with hydrated amorphous Fe‐O‐P environments, linking precipitation and washing for lithium management in lithium iron phosphate recycling.

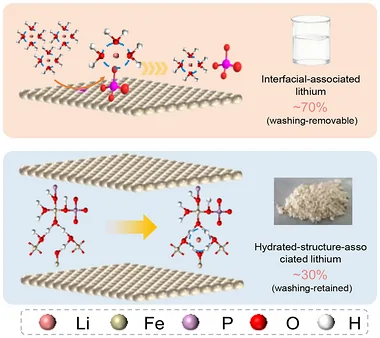

## 1 Introduction

The rapid expansion of the electric vehicle and energy storage industries has led to a substantial increase in retired lithium‐ion batteries [1, 2, 3]. In China, end‐of‐life (EOL) power battery volumes are projected to increase dramatically from 98.9 kt in 2020 to 5.48–7.07 Mt by 2030, resulting in cumulative generation of 16.67–19.99 Mt over 2020–2030 [4]. Among various battery chemistries, lithium iron phosphate (LiFePO_4_, LFP) batteries have become increasingly dominant due to their low cost, high safety, and long cycle life, accounting for 60.10% of the total retired battery mass in China by 2030 under the baseline scenario [4, 5, 6, 7]. Therefore, efficient recycling of spent LFP batteries is essential for lithium resource recovery [6, 8, 9, 10]. Hydrometallurgy remains the primary recycling route, involving leaching, separation, purification, and regeneration [11, 12, 13]. Although optimized leaching achieves > 95% lithium extraction, subsequent separation processes are accompanied by lithium migration and loss, limiting resource utilization [13, 14, 15]. Understanding lithium migration pathways and mechanisms is therefore critical to improving lithium recovery from spent LFP batteries.

In subsequent separation and regeneration processes, the oxidative precipitation of FePO_4_ is a critical step for Fe/Li separation and an important stage at which lithium loss or carryover may occur [16, 17, 18]. Although lithium is generally expected to remain predominantly in the aqueous phase under acidic conditions, measurable amounts of lithium are frequently detected in regenerated FePO_4_ products [16, 19, 20, 21]. Previous studies have extensively investigated lithium extraction efficiency and FePO_4_ regeneration during spent LFP recycling [22, 23, 24, 25]. Lou et al. revealed that FePO_4_ precipitation involves an amorphous FePO_4_·xH_2_O intermediate, highlighting the structural evolution associated with this process [26]. Jing et al. developed a chemical precipitation strategy for the simultaneous recovery of FePO_4_ and Li_2_CO_3_ from spent LFP leachates [27]. However, these studies primarily evaluated lithium recovery efficiency or FePO_4_ product quality, without addressing how lithium is retained during FePO_4_ precipitation or whether such retention arises from specific interactions with the precipitated phase. Jiang et al. proposed that lithium ions are unlikely to enter the FePO_4_ lattice under acidic oxidative precipitation conditions [28]. Conversely, Vasconcelos et al. suggested that residual lithium in regenerated FePO_4_ may originate from physical retention or coprecipitation trapping of lithium species during precipitation [16]. These explanations were mainly based on indirect observations, and the specific lithium migration pathways, retention environments, and persistence mechanisms during FePO_4_ precipitation remain unclear. Therefore, understanding lithium redistribution during FePO_4_ precipitation is essential for developing targeted strategies to minimize lithium carryover and improve lithium resource utilization in closed‐loop LFP recycling [29].

Here, we systematically investigate lithium partitioning and possible local retention environments during FePO_4_ regeneration from spent LFP cathodes. To distinguish intrinsic retention associated with FePO_4_ formation from retention under practical recycling conditions, three representative systems are examined: a simulated Fe–P system, a commercial LFP‐derived leachate, and a spent black mass‐derived leachate. Lithium retention is examined by linking its partitioning and washing behavior to precipitation and structural evolution. The results distinguish two fractions by washing accessibility and support a proposed model involving Li association with phosphate‐containing interfaces and internal hydrated amorphous Fe–O–P environments. This framework links lithium carryover to precipitation and washing, provides a basis for accounting for precursor‐retained Li, and offers strategies to regulate these two steps in closed‐loop LFP resynthesis.

## 2 Results and Discussion

### 2.1 Lithium Redistribution During FePO_4_ Regeneration

Regeneration of FePO_4_ from acidic LFP leachates is intended to recover iron and phosphorus in a solid precursor while retaining lithium in solution [30]. The process examined here comprises oxidative precipitation, solid–liquid separation, sequential washing, and drying (Figure 1a). Most lithium remained in the liquid phase, but measurable lithium was detected in the FePO_4_ products after separation and washing. For FP‐B and FP‐C, Li leaching efficiencies were 98.50% and 98.11%, respectively, while only approximately 0.45% and 0.23% of the initial feed Li remained in the washed precipitates (Table S3).

**FIGURE 1:**
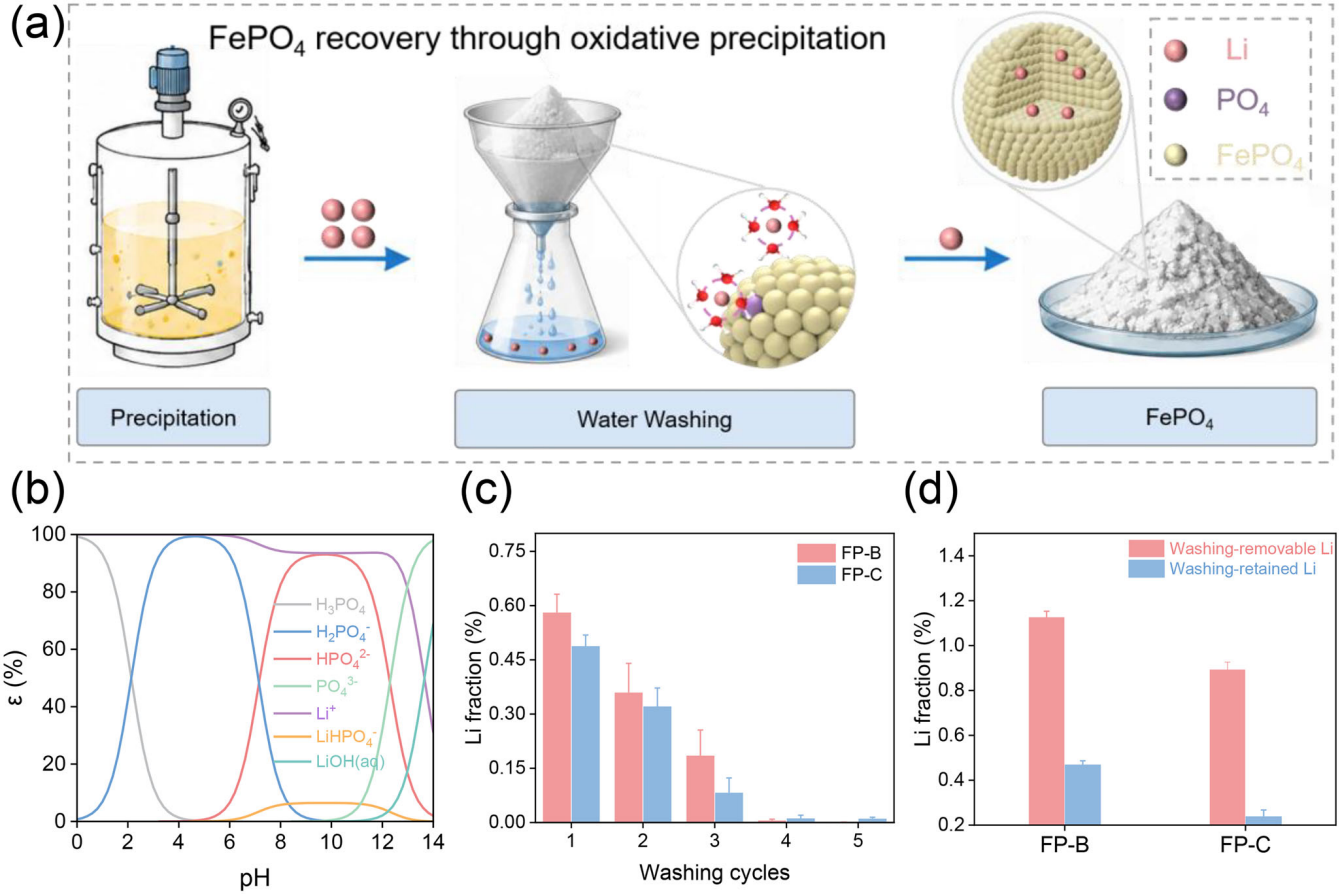
Identification of lithium retention during FePO_4_ recovery. (a) Schematic of the FePO_4_ recovery process from spent LFP cathodes. (b) pH‐dependent speciation of phosphate‐ and lithium‐containing species. (c) Lithium release patterns during sequential washing of the recovered FePO_4_ precipitates. (d) Distribution of lithium between the washing solutions and the washed FePO_4_ products.

To clarify the origin of the retained lithium, we first evaluated the distribution of aqueous species under the precipitation conditions. As illustrated in Figure 1b, within the investigated pH window of 1–3, phosphate is present predominantly as H_3_PO_4_ and H_2_PO_4_^−^, with their relative proportions shifting systematically as pH increases [31]. Within the idealized aqueous speciation shown in Figure 1b, Li^+^ is the dominant dissolved lithium species, whereas LiHPO_4_^−^ and LiOH contribute negligibly [32]. Thermodynamic calculations likewise indicate that the formation of the evaluated lithium‐containing solid phases is thermodynamically disfavored under the modeled conditions [33]. Taken together, these results suggest that the lithium detected in regenerated FePO_4_ may be associated with the evolving precipitate environment.

The washing behavior of the retained lithium was subsequently examined through sequential washing experiments. As shown in Figure 1c and Table S4, the amount of lithium released into the collected wash solutions declined with successive washing cycles in both FP‐B and FP‐C. For FP‐B, it decreased from 0.58% in the first cycle to 0.36% and 0.19% in the second and third cycles, respectively; the corresponding values for FP‐C were 0.49%, 0.32%, and 0.08%. Li release decreased substantially in the fourth and fifth washing cycles for both FP‐B and FP‐C. The relatively high lithium release during the initial washing step implies that a substantial proportion of the retained lithium remains readily accessible to water and can be easily removed. This wash‐released lithium may originate from residual acid leachate associated with the precipitates or from readily accessible interfacial environments [19, 27]. In contrast, the progressively diminished lithium release in later washing cycles points to a lithium fraction that is more difficult to access within the recovered FePO_4_ precipitates. Extending the sonication time from 3 to 7 min increased the washing‐removable Li fraction in both FP‐B and FP‐C, while Li remained in the washed solids at each tested duration (Figure S4). Together, these results identify Li fractions with different washing accessibility under the conditions examined.

The influence of precursor composition on lithium retention was further probed using FePO_4_ precipitates obtained from commercial LFP‐derived leachate (FP‐B) and spent black mass‐derived leachate (FP‐C) (Figure 1d). In both systems, lithium exhibited similar release behavior: most of the associated lithium was removed during washing, whereas a smaller fraction remained in the washed FePO_4_ products. Under otherwise comparable precipitation conditions, however, FP‐B contained higher amounts of both washing‐removable and washing‐retained lithium than FP‐C. This systematic difference suggests that the chemical composition of the precursor solutions may influence lithium redistribution during FePO_4_ formation [17]. In particular, the different Li, Fe, P, and impurity contents of the two leachates (Table S2) can modify the local precipitation environment and thereby modulate lithium retention [34]. Taken together, these results demonstrate that lithium retention occurs in parallel with FePO_4_ precipitation, even though lithium remains predominantly dissolved in the acid leachate under acidic conditions. The coexistence of lithium fractions with different washing accessibility is consistent with multiple retention environments in the recovered FePO_4_ precipitates.

### 2.2 Precipitation‐Controlled Lithium Partitioning

To clarify how precipitation conditions govern lithium redistribution, we systematically evaluated the effects of pH and temperature on Li partitioning (Figure 2a and Figure 2b). When the pH was raised from 1.0 to 3.0, Fe precipitation increased markedly, reaching 99.8% for FP‐B and 99.59% for FP‐C. Notably, the behaviors of both the washing‐removable and washing‐retained Li fractions diverged from the pattern of Fe precipitation efficiency [35]. As pH increased from 1.0 to 2.0, both Li fractions rose, but the washing‐removable fraction declined upon further elevation of pH beyond 2.0. This non‐monotonic response suggests that lithium redistribution is not governed solely by the overall extent of FePO_4_ precipitation; instead, it is also associated with the local physicochemical environment developed during the precipitation process [36].

**FIGURE 2:**
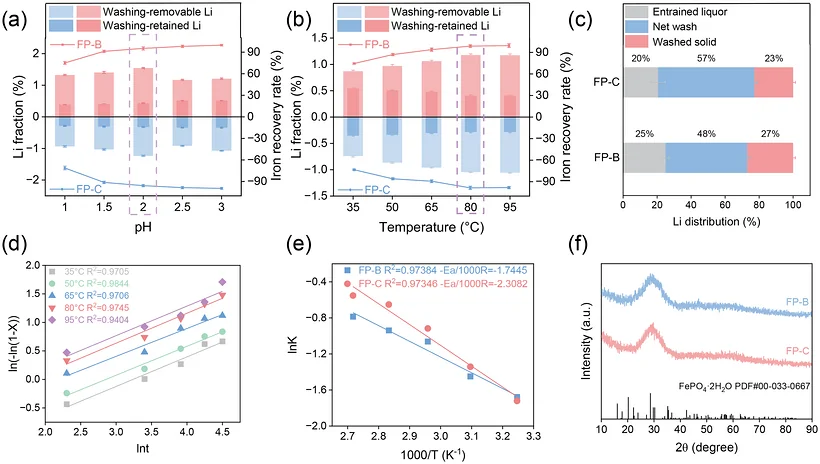
Precipitation‐dependent lithium redistribution in FP‐B and FP‐C systems. (a) pH‐dependent Fe precipitation efficiency and lithium partitioning. (b) Temperature‐dependent Fe precipitation efficiency and lithium partitioning. (c) Relative contributions of Li attributable to entrained mother liquor, net washing‐released Li, and Li retained in the washed solids to the total precipitate‐associated Li in FP‐B and FP‐C under representative precipitation conditions. (d) Avrami fits of time‐dependent FePO_4_ precipitation at different temperatures. (e) Arrhenius‐form regressions of *ln k* against *1000/T* used to obtain formal *Ea* values. (f) XRD patterns of recovered FePO_4_ precipitates.

Temperature further regulated the partitioning of retained lithium during FePO_4_ precipitation. As the temperature increased from 35°C to 95°C, Fe precipitation increased from 74.44% to 98.80% in FP‐B and from 73.33% to 98.58% in FP‐C, accompanied by a decrease in the washing‐retained fraction from 0.54% to 0.40% and from 0.35% to 0.28%, respectively. This opposite trend indicates that enhanced Fe precipitation does not simply promote overall lithium retention, but instead alters the distribution of lithium between the washing‐removable and washing‐retained fractions. Together, the pH and temperature results show that Fe precipitation efficiency alone does not determine Li partitioning.

Under representative precipitation conditions, approximately 73% and 77% of the total precipitate‐associated lithium in FP‐B and FP‐C, respectively, was removed during washing. Part of this washing‐removable lithium was estimated to be attributable to entrained mother liquor, with Li fractions of approximately 0.47% in FP‐B and 0.24% in FP‐C (Figure S6). As shown in Figure 2c, entrained mother liquor was estimated to account for 25% and 20% of the total precipitate‐associated lithium in FP‐B and FP‐C, respectively. After subtracting this contribution, net washing‐released lithium accounted for approximately 48% and 57%, whereas washing‐retained lithium accounted for approximately 27% and 23%. These proportions demonstrate that the majority of the retained lithium is in a fraction removable by washing, whereas a smaller fraction exhibits reduced accessibility within the recovered precipitates. The effects of hydrogen peroxide (H_2_O_2_) dosage and reaction time further clarify the relationship between lithium retention and FePO_4_ formation. As depicted in Figure S5b, decreasing the H_2_O_2_ dosage reduced iron precipitation to approximately 70%–75% and decreased both lithium fractions, with a disproportionately larger decrease in the washing‐retained fraction. H_2_O_2_ dosage directly controls Fe^2+^ oxidation and subsequent FePO_4_ precipitation. The heightened sensitivity of washing‐retained lithium to this parameter suggests that this fraction is linked to FePO_4_ formation. Extending the reaction time increased iron precipitation and progressively stabilized lithium partitioning (Figure S5a).

We fitted the time‐dependent Fe precipitation fractions of FP‐B and FP‐C using the Avrami equation [37]:

(1) $$\alpha =1-exp\left(-kt^{n}\right)$$

The equation was linearized as:

(2) $$\ln\left[-\ln\left(1-\alpha \right)\right]=\ln k+n\ln t$$

where *α* denotes the fraction of Fe precipitated at time *t*, *k* is the fitted precipitation coefficient, and *n* is the Avrami exponent describing the time dependence [38]. The linearized fits gave R^2^ values of 0.9404–0.9909 (Figure 2d, Figure S7 and Table S7). In both systems, n remained close to 0.5 across the temperature range, while *k* increased from 0.1867 to 0.4555 for FP‐B and from 0.1788 to 0.5755 for FP‐C. The similar exponents indicate comparable shapes of the precipitation time courses, whereas the increase in *k* reflects faster precipitation at elevated temperature. We then evaluated the temperature dependence of *k* using the Arrhenius equation:

(3) $$\ln k=\ln A\;-\frac{E_{a}}{R}\cdot \frac{1}{T}$$

where *A* represents the pre‐exponential factor, *Ea* is the formal activation energy, *R* is the universal gas constant, and *T* is the absolute temperature [39]. For the plots of *ln k* against *1000/T*, the slope is −*Ea/(1000R)*, yielding *Ea* values of 14.50 ± 1.37 kJ mol^−1^ for FP‐B and 19.19 ± 1.83 kJ mol^−1^ for FP‐C (Figure 2e and Table S7; fitting details are provided in Text S3). Both systems therefore show a temperature‐driven increase in the fitted precipitation coefficient, with a numerically stronger temperature response for FP‐C. Their near‐constant Avrami exponents indicate broadly comparable macroscopic precipitation behavior, although this need not imply identical microscopic pathways [40]. Because FP‐B and FP‐C nevertheless retain different amounts of lithium, the local chemical environments formed during precipitation also appear important for lithium partitioning.

XRD analysis was further conducted to elucidate the structural characteristics of the recovered products. As shown in Figure 2f, FP‐B and FP‐C both display broad diffraction features without discernible sharp crystalline reflections, indicating that the recovered FePO_4_ products are predominantly amorphous. No crystalline Li‐containing phase was detected within the sensitivity of XRD, but this observation cannot exclude low‐content, poorly crystalline, or amorphous Li‐bearing species [41]. The possible roles of local hydration and interfacial structure are examined using complementary measurements in the following sections [42].

### 2.3 Interfacial Environments Associated with Lithium Retention

Although lithium redistribution during FePO_4_ regeneration has been established, whether the retained lithium originated from discrete lithium‐containing phases remained unresolved. We therefore examined the thermodynamics of the precipitation system. The E–pH diagram shows that the experimental conditions fall within the stability domain of FePO_4_ (Figure 3a) [43]. Consistent with this result, H_3_PO_4_ and H_2_PO_4_^−^ dominate the aqueous phosphate speciation over the investigated pH range, whereas the calculated PO_4_^3−^ fraction remains negligible (Figure 3b). Gibbs free‐energy calculations further indicate that FePO_4_ formation has a substantially greater thermodynamic driving force than the evaluated lithium phosphate reactions (Figure 3c), while competing reactions involving impurity ions are also unfavorable under the applied conditions (Figure S9).

**FIGURE 3:**
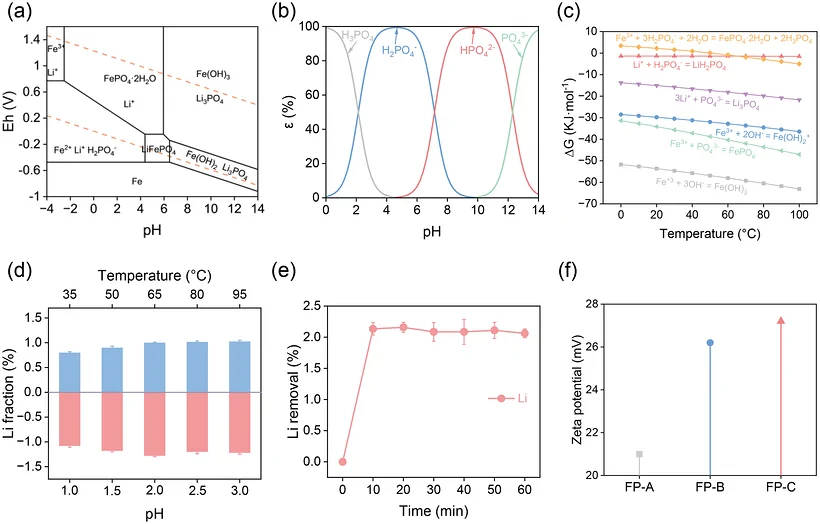
Thermodynamic and interfacial evidence relevant to lithium retention during FePO_4_ precipitation. (a) E‐pH diagram of the LFP‐derived system. (b) Phosphate speciation as a function of pH. (c) Gibbs free‐energy changes for the evaluated precipitation reactions. (d) P‐based estimated Li fractions under different pH and temperature conditions, assuming Li/p = 3 (Text S1). Red bars are inverted for visual clarity. (e) Time‐dependent removal of dissolved Li by preformed Li‐free FP‐A. (f) Zeta potentials of FP‐A, FP‐B, and FP‐C.

The idealized Fe–H_2_O speciation diagram suggests extensive Fe(III) hydrolysis (Figure S8); however, the sulfate‐rich composition of the actual process liquor introduces additional complexation equilibria. Conditional PHREEQC calculations of the post‐precipitation hot filtrates predicted that a larger fraction of the residual dissolved Fe(III) was sulfate‐complexed at higher temperature. For example, the calculated fraction of FeSO_4_^+^ in FP‐B increased from 6.53% at 35°C to 75.98% at 95°C (Text S2 and Tables S8–S10), consistent with a redistribution of dissolved Fe(III) away from free Fe^3+^. The predicted Li–sulfate association likewise represents aqueous ion pairing rather than the precipitation of a separate lithium‐containing solid, although its magnitude was sensitive to the thermodynamic database used (Table S11). Because these calculations describe only the terminal hot filtrates, they cannot reconstruct the earlier saturation states or independently identify the recovered solid phase. Taken together, these calculations suggest that the evaluated phosphate phases may be unfavorable under the modeled conditions. Solution speciation may also influence Li retention during FePO_4_ precipitation.

(4) $$Fe^{3+}+3H_{2}PO_{4}^{-}+2H_{2}O=FePO_{4}\cdot 2H_{2}O+2H_{3}PO_{4}$$

(5) $$Li^{+}+H_{2}PO_{4}^{-}=LiH_{2}PO_{4}$$

(6) $$3Li^{+}+P\;O_{4}^{3-}=Li_{3}PO_{4}$$

(7) $$Fe^{3+}+PO_{4}^{3-}=FePO_{4}$$

(8) $$Fe^{3+}+2OH^{-}=Fe{\left(OH\right)}_{2}^{+}$$

(9) $$Fe^{3+}+3OH^{-}=Fe{\left(OH\right)}_{3}$$

To assess whether phosphorus release could account for the Li released during washing, we calculated P‐based Li estimates using the measured total phosphorus in the washing solutions (Figure S10). The calculations used the assumed Li/P molar ratios defined in Text S1. The estimates under different pH and temperature conditions are presented in Figure 3d. Under most investigated conditions, these estimates were lower than the measured total Li released during washing, even at Li/p = 3 (Figure 2a,b). Within the assumed Li/P range, this difference indicates that the fixed‐ratio estimate does not describe all Li released during washing. To examine a possible contribution from FePO_4_ itself, we next tested Li association with preformed Li‐free FP‐A.

XRD patterns of the lithium‐free FP‐A sample are shown in Figure S11. This sample was further used to investigate the lithium‐interception behavior of FePO_4_ precipitates. When preformed Li‐free FP‐A was immersed in Li_2_SO_4_ solution, the dissolved Li concentration decreased by 2.13% relative to its initial value and approached a plateau within 10 min (Figure 3e and Text S5). The apparent decrease in dissolved Li concentration is consistent with Li uptake by preformed Li‐free FP‐A under the tested contact conditions [44, 45]. Following contact, Li release decreased progressively during five successive water‐washing cycles and became minimal in the fifth wash (Figure S13), indicating that part of the Li associated with preformed FP‐A was accessible to water washing. These observations suggest a possible contribution of FePO_4_‐associated uptake to lithium carryover during regeneration.

The interfacial properties of the FePO_4_ precipitates were examined in greater detail to clarify their relationship to lithium retention. FP‐A, FP‐B, and FP‐C displayed positive zeta potentials of 21.0, 26.2, and 27.2 mV, respectively (Figure 3f) [46]. The positive zeta potentials measured at pH 2 align with the expected protonation of FePO_4_ surfaces formed under acidic precipitation conditions [41]. Notably, the progressive increase in zeta potential across the three FePO_4_ systems suggests differences in their interfacial chemical environments during precipitation, which may underlie the distinct lithium retention behaviors observed. We also prepared Li‐dosed FP‐A under varied precipitation conditions to examine Li retention when Li was present during FePO_4_ formation (Figure S12). Variations in pH, temperature, and reaction time affected both Li fractions (Figure S12a–c). In Figure S12d, washing‐removable and washing‐retained Li accounted for 1.71% and 0.46% of the initial Li input, respectively. These trends are consistent with a role for precipitation conditions in Li retention across systems with different precursor compositions.

Collectively, these findings suggest that developing phosphate‐containing interfaces may contribute to Li retention during FePO_4_ precipitation, while no discrete crystalline Li‐containing phase was detected by XRD. The persistence of part of the Li after washing led us to examine the water‐associated properties and internal environments of the precipitates.

### 2.4 Hydrated Amorphous Environments Associated with Washing‐Retained Lithium

Lithium remaining after three washes represents a less readily removable fraction of the recovered precipitates. We compared the water‐associated properties and local Fe–O–P environments of FP‐A, FP‐B, and FP‐C.

Thermogravimetric analysis revealed an initial mass‐loss region shared by FP‐A, FP‐B, and FP‐C, followed by more distinct behavior at higher temperatures (Figure 4a). All three samples lost approximately 20% of their initial mass by 200°C, indicating that this early water‐related loss was not specific to Li retention in the precipitate [47]. Their DTG profiles nevertheless differed (Figure S14): FP‐A showed a weak feature near 51°C, whereas FP‐B and FP‐C displayed more pronounced dehydration responses, with FP‐C exhibiting the sharpest peak near 103°C. From approximately 200°C to 400°C, the additional mass losses were about 5%, 14%, and 12% for FP‐A, FP‐B, and FP‐C, respectively. The resulting cumulative losses at approximately 400°C were 25.3%, 33.2%, and 31.1%. Thus, FP‐C had the sharpest low‐temperature DTG peak, but FP‐B underwent the greatest cumulative mass loss. FP‐A was synthesized from a Li‐free Fe–P solution, FP‐B was derived from low‐impurity commercial LFP, and FP‐C represented the more complex spent‐LFP leachate (Tables S1 and S2). The FP‐A–FP‐B comparison shows that the greater higher‐temperature mass loss in FP‐B also occurred at relatively low co‐leached metal levels. This comparison is relevant to recycling flowsheets in which impurity removal precedes FePO_4_ precipitation. FP‐B also retained more Li after washing than FP‐C. These comparisons suggest an association between washing‐retained Li and the overall water‐related mass‐loss profile, rather than the height of a single dehydration peak.

**FIGURE 4:**
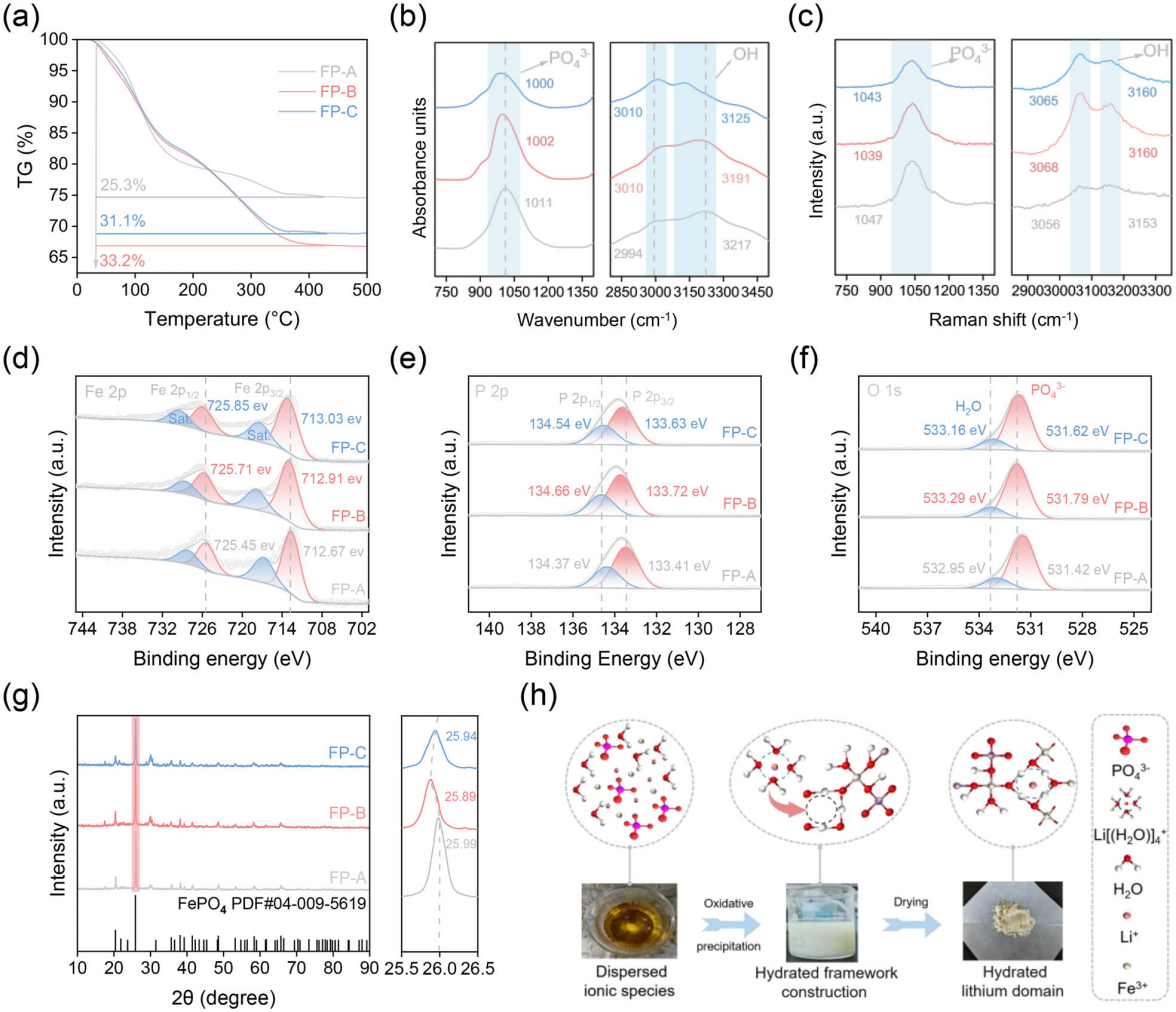
Hydration and local‐structure evidence associated with washing‐retained lithium in amorphous FePO_4_. (a) TGA profiles of FP‐A, FP‐B, and FP‐C. (b) FTIR spectra and (c) Raman spectra of phosphate‐ and water‐related features. (d–f) XPS spectra of the Fe 2p, P 2p, and O 1s regions. (g) XRD patterns after calcination. (h) Proposed association of lithium retention with hydrated local environments.

FTIR analysis further revealed differences in phosphate and hydroxyl environments (Figure 4b and Figure S15). In the FTIR spectra, the characteristic phosphate vibrational band, originally at 1011 cm^−1^ in FP‐A, shifted to 1002 cm^−1^ in FP‐B and 1000 cm^−1^ in FP‐C, corresponding to red shifts of 9 and 11 cm^−1^, respectively. These systematic shifts indicate a modified local phosphate environment among the recovered precipitates. Concomitantly, the O–H vibrational region underwent marked changes. The lower‐frequency band, at 2994 cm^−1^ in FP‐A, moved to 3010 cm^−1^ in both FP‐B and FP‐C, while the higher‐frequency component shifted from 3217 to 3191 and 3125 cm^−1^, respectively. The more pronounced shift observed for FP‐C may reflect stronger hydrogen‐bonding interactions and a more heterogeneous water‐associated environment.

To further assess whether hydration is accompanied by local structural evolution, Raman spectroscopy was used to monitor changes in phosphate and hydroxyl environments (Figure 4c and Figure S16). The PO_4_ stretching mode shifted from 1047 cm^−1^ in FP‐A to 1039 cm^−1^ in FP‐B and 1043 cm^−1^ in FP‐C, indicating modified phosphate‐oxygen environments among the recovered precipitates. Concomitantly, hydroxyl‐related bands displayed systematic shifts: the characteristic features at 3056 and 3153 cm^−1^ in FP‐A moved to 3068 and 3160 cm^−1^ in FP‐B, and to 3065 and 3160 cm^−1^ in FP‐C, respectively. Although the extent of these spectral changes differed between FP‐B and FP‐C, reflecting their distinct precursor compositions, both lithium‐containing systems showed consistent deviations relative to FP‐A. Together, these shifts show that phosphate‐ and water‐related environments differ among FP‐A, FP‐B, and FP‐C. Their correspondence with retained Li supports the proposed hydrated‐environment interpretation.

The local chemical environments within the Fe–O–P framework were further probed by XPS analysis (Figures 4d–f). The Fe 2p spectra show that all recovered precipitates retain the characteristic Fe^3+^ oxidation state, indicating that no major change in the measured Fe oxidation state accompanies lithium retention. Despite this invariance in oxidation state, discernible shifts in binding energy are observed across the precipitates. Specifically, the Fe 2p_3/2_ peak shifts from 712.67 eV in FP‐A to 712.91 eV in FP‐B and 713.03 eV in FP‐C, while the corresponding Fe 2p_1/2_ peak shifts from 725.45 to 725.71 and 725.85 eV, respectively. These positive shifts are consistent with alterations in the local Fe–O coordination environment associated with structural evolution. Similar trends are observed in the P 2p spectra. The P 2p_3/2_ peak shifts from 133.41 eV in FP‐A to 133.72 eV in FP‐B and 133.63 eV in FP‐C, and the P 2p_1/2_ component shifts from 134.37 to 134.66 and 134.54 eV, respectively. Taken together, these variations indicate that phosphate groups experience modified electronic environments during lithium‐associated precipitation. The O 1s spectra further reveal concerted variations in both phosphate oxygen and water‐associated oxygen species. Specifically, the phosphate oxygen component shifts from 531.42 eV in FP‐A to 531.79 eV in FP‐B and 531.62 eV in FP‐C, while the water/hydroxyl‐related component shifts from 532.95 to 533.29 and 533.16 eV, respectively. Together, the Fe, P, and O changes are consistent with an association between washing‐retained Li and evolving hydrated Fe–O–P environments, without by themselves excluding physical retention within the precipitates.

XRD analysis was conducted on the calcined products to ascertain whether the washing‐retained lithium species facilitated the development of additional Li‐containing crystalline phases during thermal treatment (Figure 4g). All samples exhibited the characteristic diffraction peaks of FePO_4_‐derived crystalline phases, and no supplementary reflections indicative of lithium‐containing impurity phases were detected. Subtle yet evident differences in diffraction patterns emerged among the samples. The principal diffraction peak at approximately 25.9° shifted from 25.99° in FP‐A to 25.89° in FP‐B and 25.94° in FP‐C, accompanied by variations in relative peak intensity. These peak shifts are consistent with minor structural differences and possible microstrain. SEM images provide complementary information on the aggregation morphology of the recovered precipitates (Figure S17). Compared with the relatively compact nanoparticle aggregates in FP‐A, FP‐B and FP‐C exhibit more irregular and heterogeneous aggregation, with more apparent interparticle spaces [48, 49]. EDS spectra and semiquantitative analyses identify Fe, P, and O as the main detected elements in all three samples. Corresponding EDS elemental maps show the spatial distributions of these elements in the imaged regions of FP‐A, FP‐B, and FP‐C (Figure S18).

Based on these observations, we propose a lithium‐retention model involving hydrated structural motifs (Figure 4h). During oxidative precipitation, nucleation and aggregation of FePO_4_·xH_2_O give rise to amorphous Fe–O–P frameworks that inherently exhibit pronounced structural heterogeneity. Within these disordered frameworks, internal hydrated environments may develop, enriched in phosphate oxygen sites and water‐associated species; these environments may contribute to Li retention. In this proposed model, the two operational fractions—washing‐removable and washing‐retained Li—may be associated with phosphate‐containing interfacial regions and internal hydrated environments of lower washing accessibility, respectively.

### 2.5 Proposed Lithium Retention Model and Process Implications

By integrating the interfacial and hydration‐related observations described above, we propose a unified lithium retention model linking lithium redistribution to precursor structural evolution during FePO_4_ regeneration (Figure 5a). During precipitation, lithium species are not removed from the system; instead, they are redistributed across different retention environments. One fraction of lithium may interact with phosphate‐containing interfaces via phosphate‐mediated interfacial association, whereas another fraction may be associated with internal hydrated, heterogeneous environments formed during amorphous Fe–O–P framework formation. In this proposed model, both retention modes arise from the same precipitation‐driven structural evolution process [47]. Consequently, lithium redistribution during FePO_4_ regeneration may involve multiple retention modes coupled to precursor structural evolution.

**FIGURE 5:**
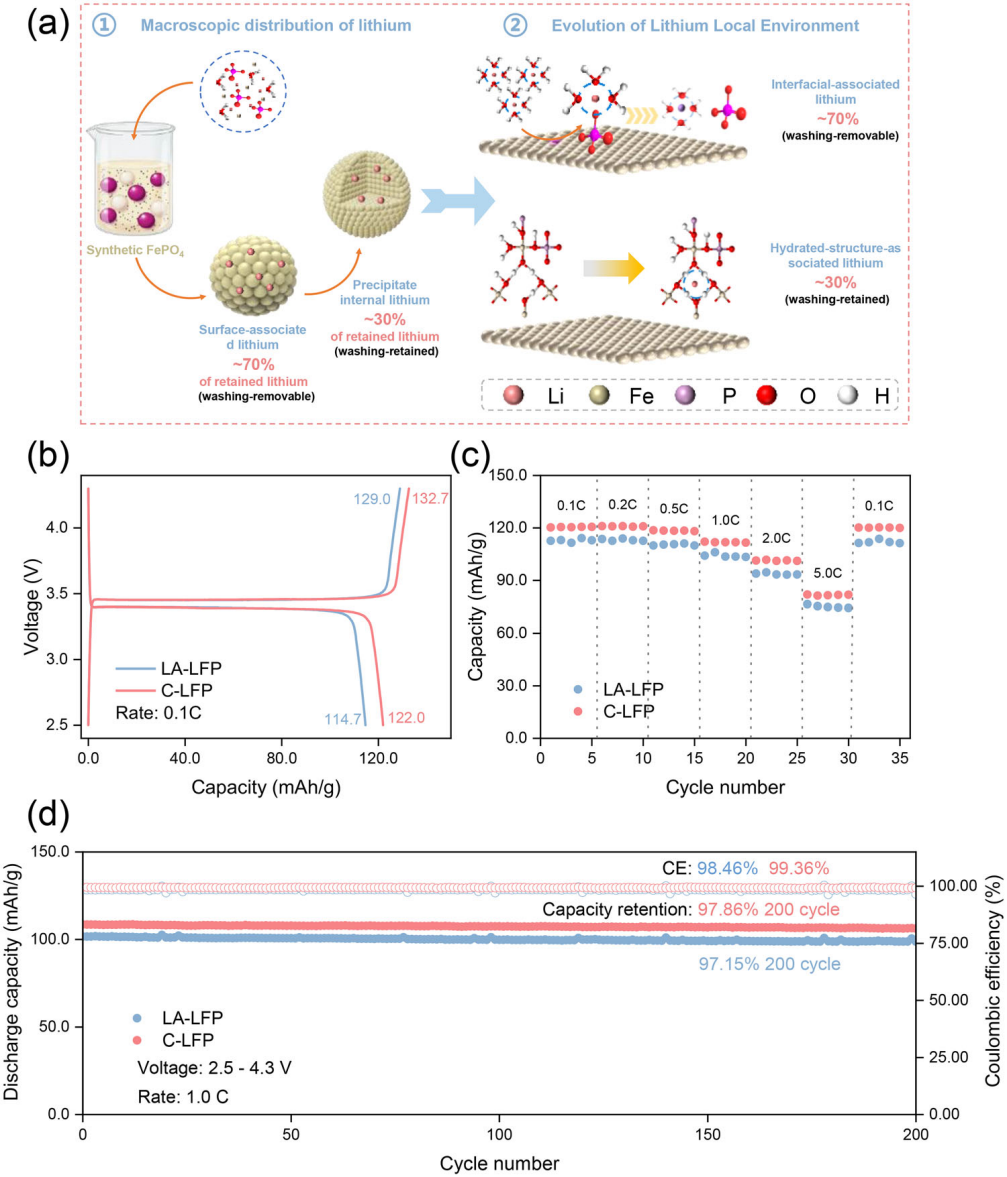
(a) Schematic of the proposed lithium redistribution model during FePO_4_ regeneration; (b) Charge/discharge curves of LA‐LFP and C‐LFP at 0.1 C; (c) Rate performance from 0.1 to 5 C and subsequent return to 0.1 C; (d) Cycling performance and Coulombic efficiency at 1 C for 200 cycles within a voltage window of 2.5–4.3 V.

The proposed retention model also has a practical implication: Li carried into the regenerated FePO_4_ precursor can be included when determining the external Li‐source dosage for LFP resynthesis (Figure S20). Lithium‐assisted LFP (LA‐LFP) was synthesized from Li‐containing FePO_4_ with reduced external Li addition, while conventional LFP (C‐LFP) prepared from Li‐free FePO_4_ served as the control (Text S6). Both samples exhibited diffraction peaks consistent with olivine LiFePO_4_, without obvious impurity reflections. The SEM insets show agglomerates of irregularly shaped particles in both samples (Figure S19). At 0.1 C, LA‐LFP delivered a discharge capacity of 114.7 mAh g^−^^1^, compared with 122.0 mAh g^−^^1^ for C‐LFP (Figure 5b). Its discharge capacity recovered to near the initial level when the rate returned from 5 C to 0.1 C (Figure 5c). After 200 cycles at 1 C, LA‐LFP and C‐LFP retained 97.15% and 97.86% of their initial capacities, respectively, with mean Coulombic efficiencies of 98.46% and 99.36% (Figure 5d). These results support accounting for precursor‐retained Li to reduce external Li addition during LFP resynthesis.

## 3 Conclusions

In summary, lithium carryover during FePO_4_ regeneration was resolved into fractions with different accessibility to washing. Under representative conditions, approximately 70% of the lithium associated with the precipitates was washing‐removable, whereas approximately 30% remained after three washing cycles. Contact with lithium‐free FePO_4_ resulted in an apparent 2.13% decrease in dissolved Li concentration, consistent with Li uptake under the tested conditions. The formation of the considered independent lithium precipitates was thermodynamically unfavorable, and no crystalline lithium‐containing phase was detected in the FePO_4_ precipitates. Differences in hydration, phosphate environments, and local Fe–O–P chemistry correlated with the washing‐retained fraction across simulated and practical systems. Together, these results support a proposed model in which lithium may be associated with phosphate‐containing interfacial groups and internal hydrated environments in amorphous FePO_4_. The model identifies precipitation and washing as linked control stages for lithium management, while the atomic‐scale lithium sites remain to be established. The LFP resynthesis results support the use of retained Li as a partial lithium source, reducing external Li addition. Further work could optimize the external Li‐source dosage and resynthesis conditions to improve lithium utilization and LFP performance.

## 4 Experimental Section

### 4.1 Materials and Reagents

The spent LFP cathode powder utilized in this study was supplied by Ganfeng Recycling Technology Co., Ltd. (Figure S1). For comparative analysis with leachates derived from spent LFP, commercial LFP powder was procured from Shanghai Titan Scientific Co., Ltd. The spent LFP batteries were first completely discharged and then subjected to mechanical crushing and sieving processes. The resulting material was subsequently thermally treated to remove organic constituents, including binders and conductive carbon, thereby producing pretreated spent LFP cathode powder. This powder was then sieved through a 200‐mesh screen before compositional analysis. To determine the elemental composition, the sample was entirely digested in aqua regia, and the resulting solution was analyzed using inductively coupled plasma optical emission spectroscopy (ICP‐OES). The elemental concentrations are summarized in Table S1. All chemicals utilized in this study were of analytical grade, and all aqueous solutions were prepared with deionized water.

### 4.2 Lithium Partitioning During FePO_4_ Regeneration

LFP powder (1 g) was leached in 30 mL of 2 M H_2_SO_4_ at 80°C for 1 h under continuous stirring at 400 rpm. After leaching, the slurry was filtered through a 0.45 µm polyethersulfone (PES) membrane to separate the solid residue from the leachate. The concentrations of Li and Fe in the leachate were measured by AAS, whereas the P concentration was determined by ICP‐OES. The measured elemental concentrations are reported in Table S2 in g L^−1^. The leaching efficiencies were calculated according to Equation (10), and the results are presented in Figure S2.

(10) $$\mu =\frac{C\times V}{m_{0}}\;\times 100\%$$

where C denotes the concentration of the target element in the leachate, V the volume of the leachate, and m_0_ the initial amount of the corresponding element in the LFP powder.

The resulting leachates were used for FePO_4_ precipitation and Li/Fe separation. Based on the measured Fe and P contents (Table S2), the initial P/Fe molar ratios were 0.972 for FP‐B and 0.778 for FP‐C. For FP‐C, 0.335 g of Na_3_PO_4_·12H_2_O was added to each 30 mL aliquot of spent‐LFP‐derived leachate, increasing the nominal P/Fe ratio to 0.972. The Fe and P mass balances for FP‐B and FP‐C, including the additional P input for FP‐C, are summarized in Table S5. The leachates were then treated with 30% H_2_O_2_ to oxidize Fe^2+^ to Fe^3+^, and the pH was adjusted with NaOH. Precipitation was carried out in an oil bath at the specified temperature and reaction time under continuous stirring. After precipitation, the solid and liquid phases were separated using a 0.45 µm membrane filter, and their Li and Fe contents were quantified. The filtrates were subsequently treated to recover Li_2_CO_3_ (Figure S3). The effects of pH (1.0–3.0), reaction time (10–90 min), temperature (35°C–95°C), and H_2_O_2_ dosage (H_2_O_2_/leachate volume ratio of 1:110–1:10) on FePO_4_ formation and lithium retention were evaluated using single‐factor experiments. The fixed parameters for each experiment are summarized in Table S6.

To elucidate lithium retention behavior during FePO_4_ recovery, three precursor systems were examined in parallel under identical precipitation conditions. A simulated Fe–P system (FP‐A) was prepared by mixing Na_3_PO_4_·12H_2_O and FeSO_4_ solutions, with the Fe and P inputs matched to those in the commercial‐LFP leachate (Table S2), providing a Li‐free Fe–P reference system. For the separate Li‐dosed simulated experiments (Figure S12), Li_2_SO_4_ was added to match the Li input of the same leachate, while the other precipitation conditions remained unchanged. The precipitate obtained from leachate of commercial LFP was designated FP‐B, whereas the precipitate produced from leachate of spent LFP black mass was labeled FP‐C.

The collected precipitate was first redispersed in 30 mL of deionized water, ultrasonicated for 5 min, and then separated by filtration. This washing protocol was performed three consecutive times, and each filtrate was collected separately for Li and P determination by ICP‐OES. Following washing, the precipitate was dried at 60°C for 12 h in a forced‐air oven. A known mass of the dried solid was then completely digested in aqua regia, and ICP‐OES was used to analyze the resulting solutions to determine the concentrations of Li and Fe. Li recovered in the first three washing solutions was defined as washing‐removable Li. Li remaining in the solid after three washes, quantified by acid digestion and ICP‐OES, was defined as washing‐retained Li. Detailed experimental and computational methods are provided in Supporting Information.

### 4.3 Material Characterizations

The phase compositions and structural characteristics of the raw materials and recovered products were analyzed by X‐ray diffraction (XRD, Philips PW 1700). The morphology and elemental distributions of the samples were examined using field‐emission scanning electron microscopy coupled with energy‐dispersive X‐ray spectroscopy (FESEM‐EDS, JEOL JSM‐7401F). The surface chemical environments and oxidation states of the constituent elements were investigated by X‐ray photoelectron spectroscopy (XPS, Kratos AXIS Ultra DLD). The functional groups and local structural features of the recovered products were characterized by Fourier transform infrared spectroscopy (FTIR, Bruker Vertex 70) and Raman spectroscopy (Renishaw in Via Reflex). Thermogravimetric analysis (TGA, TA Instruments Q50) was performed to evaluate the thermal decomposition behavior and water‐associated structural characteristics of the precipitates. The surface charge properties and colloidal stability of the precipitates were determined using a Zetasizer Nano ZS (Malvern Instruments). The concentrations of dissolved metal ions were quantified using inductively coupled plasma optical emission spectroscopy (ICP‐OES, PerkinElmer OPTIMA 8300) and atomic absorption spectroscopy (AAS, Analytik Jena ContrAA 700). Aqueous phosphate and lithium speciation was calculated using Visual MINTEQ 3.1, while E–pH diagrams and reaction Gibbs free energies were calculated using HSC Chemistry 9.0. PHREEQC was used to evaluate ion activities, sulfate complexation, and saturation indices in the post‐precipitation hot filtrates.

## Author Contributions

**Fei Han**: conceptualization, data curation, visualization, methodology, Writing – original draft, Writing – review and editing. **Langyi Xiao**: visualization, conceptualization. **Genping Yi**: conceptualization, supervision, writing – review and editing. **Tian Liu**: methodology, investigation, writing – review and editing. **Shoudong Huang**: conceptualization. **Haonan Yan**: methodology, visualization. **Guang Yang**: writing – review and editing, conceptualization. **Liming Yang**: writing – review and editing, supervision, resources, funding acquisition. **Xubiao Luo**: visualization, conceptualization.

## Conflicts of Interest

The authors declare no conflicts of interest.

## Supporting information




**Supporting File**: advs78485‐sup‐0001‐SuppMat.docx.

## Data Availability

The data that support the findings of this study are available in the supplementary material of this article.
