# Supplementary material for: Interfacial and Hydration‐Associated Lithium Retention During FePO4 Regeneration from Spent LiFePO4 Cathodes
Source: Adv Sci (Weinh). 2026 Oct 26:e78485. Online ahead of print. doi: 10.1002/advs.78485 (PMC13710836; doi:10.1002/advs.78485)
Supplement: Supplementary file 1 [file ADVS-9999-e78485-s001.docx]

**Supplementary material**

**Interfacial and Hydration-Associated Lithium Retention during FePO_4_ Regeneration from Spent LiFePO_4_ Cathodes**

Fei Han^a,c^, Langyi Xiao^b^, Genping Yi^a,*^, Tian Liu^a^, Shoudong Huang^a^, Haonan Yan^a^, Guang Yang^a,*^, Liming Yang^a,*^, Xubiao Luo^a,b,c^

*^a^ School of Life Sciences, Key Laboratory of Jiangxi Province for Functional Biology and Pollution Control in Red Soil Regions, Jinggangshan University, Ji'an, 343009, China*

*^b^ National-Local Joint Engineering Research Center of Heavy Metals Pollutants Control and Resource Utilization, Nanchang Hangkong University, Nanchang 330063, China*

*^c^ School of Materials Science and Engineering, Beihang University, No. 37, XueYuan Road, Haidian District, Beijing 100191, China*

* Corresponding author, E-mail address: [genpingyi@126.com](mailto:genpingyi@126.com) (G. P. Yi); [guangyang@ahau.edu.cn](mailto:guangyang@ahau.edu.cn) (G. Yang); <yangliming0809185@126.com> (L. M. Yang)

**Supplementary Texts**

**Text S1 Conditional estimation of lithium associated with phosphorus released during washing**

The total amount of phosphorus in the washing solutions was calculated from the phosphorus concentration measured in each washing step:

$$\begin{aligned} n_{P,wash} = \sum_{j}\frac{C_{P,j} V_{j}}{M_{P}}\#\left( \text{1} \right) \end{aligned}$$

where *C_(P, j)_* and *V_(j)_* are the measured phosphorus concentration and solution volume in washing step *j*, respectively, and *M_(P)_* is the molar mass of phosphorus. ICP-OES measures total phosphorus in the washing solution and does not establish that it exists as free PO_4_^3-^ under the washing conditions.

To provide a conditional estimate of the lithium that could accompany the phosphorus released during washing, hypothetical Li/P molar ratios of 1, 2, and 3 were applied. The estimated lithium mass was normalized to the measured total lithium mass in the leachate *before precipitation*:

$$\begin{aligned} f_{Li,k} = \frac{k n_{P,wash} M_{Li}}{C_{Li,0} V_{0}} \times100\%\#\left( \text{2} \right) \end{aligned}$$

where *k* is the assumed Li/P molar ratio (1, 2, or 3), *M_(Li)_* is the molar mass of lithium, and *C_(Li,0)_* and *V_(0)_* are the measured lithium concentration and total volume of the leachate before precipitation. Concentrations, volumes, and molar masses were expressed in consistent units. The *k=3* case provides a deliberately generous benchmark *within this phosphorus-based estimation*. These ratios are not measured Li–phosphate coordination numbers, and the estimate does not bound Li retained through solution entrapment or other surface-associated processes.

**Text S2 Thermodynamic Speciation Analysis**

PHREEQC calculations were performed for the hot-filtered liquors collected after precipitation at 35, 50, 65, 80, and 95 °C. The pH measured in each hot filtrate was used at its corresponding reaction temperature. The reported Li, Fe, and P amounts were converted to solution concentrations using an assumed final liquid volume of 39.2 ml. Fe was entered as Fe (III) following H_2_O_2_ addition. Sodium and sulfate inputs were estimated from the reagent additions, with all added sulfate assumed to remain in solution; no constituent was adjusted to impose charge balance. The SIT database was used for the main aqueous-speciation calculations. The same solution inputs were independently recalculated with the WATEQ4F, MINTEQ v4, and LLNL databases to assess the sensitivity of Li–sulfate association to database selection.

The hot-filtrate calculations indicate that sulfate complexation affects the speciation of the Fe remaining after precipitation (Tables S9–S10). In FP-B, the calculated fraction of dissolved Fe(III) present as FeSO_4_^+^ increased from 6.53% at 35 °C to 75.98% at 95 °C. The three databases that include LiSO_4_^−^ also predict aqueous Li–sulfate association, with fractions of 23.51–47.16% for FP-B at 35 °C (Table S11). LiSO_4_^−^ is an aqueous ion pair; its predicted abundance depends strongly on database selection and does not indicate precipitation of a lithium sulfate solid. All saturation indices describe the terminal hot filtrates, so they cannot establish the saturation state earlier in the reaction. The indices for FePO_4_·2H_2_O also depend on the selected solid-phase reference constants and cannot identify the recovered solid phase. Experimental Fe and P removal remains the principal evidence for precipitation. Several solution inputs were estimated, three of the ten filtrates had absolute charge-balance errors above 10%, and the calculated ionic strengths ranged from 3.06 to 4.88 mol kg^−1^ (Table S8). The calculated activities and saturation indices should therefore be interpreted as conditional model estimates under the stated inputs.

**Text S3 Kinetic fitting and parameter interpretation**

The Fe precipitation fractions measured at 10, 30, 50, 70, and 90 min were fitted using *ln[−ln(1 − α)] = ln k + n ln t*, with *t* in minutes. The intercept gives *ln k*, the slope gives *n*, and *k = exp(ln k)* has units of min^-n^. Table S7 reports the parameter standard errors and R^2^ values of these transformed five-point fits; the standard errors describe fitting uncertainty rather than variation among independent experiments. Within each system, *n* remained approximately constant across the investigated temperatures, whereas *k* increased. The five *ln k* values were then regressed against *1000/T* (*T* in kelvin), for which the slope *b* equals −*Ea*, formal/(1000R). The reported uncertainty in *Ea*, formal was calculated from the standard error of *b* without propagating uncertainty from the individual *ln k* fits. Because *n* varies slightly with temperature, changing the time unit would also change the fitted *k* values and their Arrhenius slope. The reported Ea is therefore a formal descriptor of the fitted temperature response, not an intrinsic activation barrier.

**Text S4 Lithium retention and washing-partition calculations**

The initial Li inventory of each leachate aliquot or Li-dosed simulated solution was calculated as

$$\begin{aligned} m_{0}=C_{0}V_{0}\#\left( 3 \right) \end{aligned}$$

where *m_0_* is the initial Li mass, and *C_0_* and *V_0_* are the Li mass concentration and volume of the solution before precipitation or contact, respectively. For the simulated FP-A experiments, *m_0_* refers to the Li actually added at a dose calculated from the commercial-LFP leachate.

The Li mass released in the ith washing cycle was calculated as

$$\begin{aligned} m_{W,i}=C_{W,i}V_{W,i}\#\left( 4 \right) \end{aligned}$$

where *m_W,i_* is the Li mass in wash i, and *C_W,i_* and *V_W,i_* are its measured Li concentration and collected solution volume, respectively; i = 1, 2, or 3.

The Li mass remaining in the entire washed precipitate was calculated from the digested solid aliquot as

$$\begin{aligned} m_{R}=C_{D}V_{D}f_{D}\frac{m_{s}}{m_{a}}\#\left( 5 \right) \end{aligned}$$

where *m_R_* is the Li mass in the entire three-times-washed precipitate; *C_D_*, *V_D_*, and *f_D_* are the measured Li concentration, final volume, and additional dilution factor of the digest, respectively; *m_a_* is the dry mass of the aliquot digested; and *m_s_* is the total dry mass of the washed precipitate. If the entire precipitate was digested, *m_s_*/*m_a_* = 1.

The Li released in each wash and the Li retained after washing, both expressed as percentages of the initial Li inventory, were calculated as

$$\begin{aligned} R_{W,i}=\frac{m_{W,i}}{m_{0}}\times100\%\#\left( 6 \right) \end{aligned}$$

$$\begin{aligned} R_{R}=\frac{m_{R}}{m_{0}}\times100\%\#\left( 7 \right) \end{aligned}$$

where *R_W,i_* is the percentage of initial Li released in wash i, and *R_R_* is the percentage remaining in the solid after three washes; *m_W,i_*, *m_R_*, and *m_0_* are defined above.

For the relative contributions shown in Figure 2c, the combined Li recovered in the washes and washed solid was used as the denominator:

$$\begin{aligned} F_{W}=\frac{\sum_{i=1}^{3} m_{W,i}}{\sum_{i=1}^{3} m_{W,i}+m_{R}}\times100\%\#\left( 8 \right) \end{aligned}$$

$$\begin{aligned} F_{R}=\frac{m_{R}}{\sum_{i=1}^{3} m_{W,i}+m_{R}}\times100\%\#\left( 9 \right) \end{aligned}$$

where *F_W_* and *F_R_* are the relative washing-removable and washing-retained contributions, respectively; the sum of *m_W,i_* over i = 1, 2, and 3 is the total Li mass in the three washes, and *m_R_* is the Li mass in the washed solid. All reported values represent the average of three independent measurements.

**Text S5 FP-A contact experiment and apparent Li removal**

For the time-dependent contact experiment in Figure 3e, Li-free FP-A was first prepared from Na_3_PO_4_·12H_2_O and FeSO_4_ under the precipitation conditions described above. The precipitate was recovered by filtration and then immersed in 30 mL of aqueous Li_2_SO_4_. The initial Li concentration was adjusted to 1.379 g L^-1^ (0.1987 mol L^-1^), matching the nominal initial Li concentration of the commercial-LFP leachate used for FP-B. Because FP-A and FP-B used matched Fe and P inputs, their theoretical FePO_4_ yields per 30 mL of solution were the same. The FP-A used for contact corresponded to 1.35 g on a dry-mass basis. This gave a nominal dry-mass-based solid-to-liquid ratio of 45.0 g L^-1^ based on the added solution volume. Unlike the Li-dosed simulated precipitation experiment in Figure S12, Li_2_SO_4_ was introduced only after FP-A formation. Contact was conducted at 80 °C under continuous stirring after the initial pH had been adjusted to 2.0 with NaOH; the pH was not subsequently readjusted, and sulfate was present as the background anion. At the specified times, liquid samples were withdrawn, separated from the solid, and analyzed for Li. The washing behavior of FP-A after contact was evaluated over five successive washing cycles (Figure S13). Three cycles were adopted for routine post-treatment because most of the readily removable Li was released during the first three washes. Each wash involved redispersion in 30 mL of deionized water, ultrasonication for 5 min, and filtration. The washed solid was then dried at 60 °C for 12 h before digestion and elemental analysis.

The apparent decrease in dissolved Li at contact time *t* was calculated as:

$$\begin{aligned} D_{Li}\left( t \right)= \frac{C_{Li,0} - C_{Li,t}}{C_{Li,0}} \times100\%\#\left( 10 \right) \end{aligned}$$

where *C_Li,0_* and *C_Li,t_* are the dissolved Li concentrations before contact and at contact time *t*, respectively. The reported 2.13% is therefore the apparent decrease in dissolved Li concentration normalized to its initial value.

**Text S6 LFP resynthesis and electrochemical characterization**

Lithium-assisted LFP (LA-LFP) and conventional LFP (C-LFP) were synthesized from Li-containing and Li-free FePO_4_ precursors, respectively. FePO_4_ was mixed with Li_2_CO_3_ and glucose as the lithium and carbon sources, respectively. For the Li-containing FePO_4_ precursor, the external Li_2_CO_3_ dosage was reduced based on its measured Li content. The mixture was dispersed in 16 mL of ethanol and ball-milled at 400 rpm for 3 h. The resulting precursor was vacuum-dried at 80 °C for 4 h and thoroughly ground. The mixture was heated from room temperature to 350 °C at 5 °C min^-1^ and held for 2 h under argon for carbonization. It was subsequently heated to 700 °C at 5 °C min^-1^ and calcined for 8 h in a tube furnace under argon.

Electrodes were prepared by mixing the synthesized LFP, acetylene black, and poly(vinylidene fluoride) (PVDF) binder at a mass ratio of 8:1:1 in N-methyl-2-pyrrolidone (NMP). The mixture was stirred for 4 h to obtain a homogeneous slurry. The slurry was coated onto clean Al foil and vacuum-dried at 60 °C for 8 h. Circular electrodes with a diameter of 12 mm were punched out, with an active material loading of approximately 1.5 mg cm^-2^. CR2025-type coin cells were assembled using lithium metal as the counter electrode and Celgard 2500 as the separator. The electrolyte consisted of 1 M LiPF_6_ dissolved in ethylene carbonate (EC), diethyl carbonate (DEC), and dimethyl carbonate (DMC) at a volume ratio of 1:1:1. Cell assembly was performed in an argon-filled glovebox, with both O_2_ and H_2_O levels maintained below 0.1 ppm. Galvanostatic charge–discharge tests of LA-LFP and C-LFP were performed at room temperature using a multichannel battery testing system (CT2001A, China). All tests were conducted within a voltage window of 2.5–4.3 V (vs. Li^+^/Li), with 1 C defined as 170 mA g⁻^1^. Charge–discharge profiles were recorded at 0.1 C. Rate performance was evaluated at 0.1, 0.2, 0.5, 1, 2, and 5 C, followed by a return to 0.1 C. Cycling performance and Coulombic efficiency were evaluated at 1 C over 200 cycles.

**Supplementary Figures**

**Figure S1.** XRD pattern of spent LiFePO_4_ cathode.

**Figure S2.** Leaching efficiency of LiFePO_4_ in H_2_SO_4_ solution.

**Figure S3.** XRD pattern of Li_2_CO_3_.

**Figure S4.** Effect of sonication time on washing-removable and washing-retained Li in FP-B (pink) and FP-C (blue).

**Figure S5.** Effect of (a) reaction time and (b) H_2_O_2_/leachate volume ratio on Fe precipitation and Li distribution.

**Figure S6.** Li content attributable to entrained mother liquor in FP-B and FP-C.

**Figure S7.** Avrami kinetic fitting of FP-C precipitation.

**Figure S8.** Species distribution diagrams for Fe-H_2_O systems.

**Figure S9.** Calculated Gibbs energy changes for the indicated phosphate-forming reactions: (a) temperature dependence and (b) values at 80 °C.

**Figure S10.** Variation of phosphate molar amount in washing solution under different (a) pH and (b) temperature conditions.

**Figure S11.** XRD pattern of the lithium‑free FP‑A sample.

**Figure S12.** Effects of (a) pH, (b) temperature, and (c) reaction time on washing-removable and washing-retained Li fractions during Li-dosed FP-A synthesis; (d) Washing-removable and washing-retained Li fractions for FP-A.

**Figure S13.** Li release during five successive water-washing cycles of preformed Li-free FP-A after contact with Li_2_SO_4_ solution.

**Figure S14.** DTG curves of FP-A, FP-B, and FP-C.

**Figure S15.** FTIR spectra of FP-A, FP-B, and FP-C.

**Figure S16.** Raman spectra of FP-A, FP-B, and FP-C.

**Figure S17.** SEM images, EDS spectra, and elemental compositions of (a) FP-A, (b) FP-B, and (c) FP-C.

**Figure S18.** SEM images and corresponding EDS elemental mappings of (a) FP-A, (b) FP-B, and (c) FP-C.

**Figure S19.** XRD patterns of C-LFP and LA-LFP, with corresponding SEM images shown in the upper and lower insets, respectively.

**Figure S20.** Conceptual scheme for using Li-containing FePO_4_ in LFP resynthesis and further optimizing the external Li-source dosage.

**Supplementary Tables**

**Table S1.** Major element contents of LFP

| Species  wt.% | **Commercial LFP cathode** | | | | **Spent LFP cathode** | | | |
| --- | --- | --- | --- | --- | --- | --- | --- | --- |
| Element | Li | 4.2 | Mn | 0.005 | Li | 2.7 | Mn | 0.301 |
|  | Fe | 36.8 | Zn | 0.003 | Fe | 26.01 | Zn | 0.006 |
|  | Ca | 0.097 | Pb | 0.001 | Ca | 0.197 | Pb | 0.001 |
|  | Mg | 0.011 | Cu | 0 | Mg | 0.019 | Cu | 0.146 |
|  | Na | 0.055 | P | 19.72 | Na | 0.081 | P | 11.379 |
|  | K | 0.059 | B | 0.021 | K | 0.075 | B | 0.018 |
|  | Si | 0.018 | Ni | 0 | Si | 0.135 | Ni | 0.744 |
|  | Al | 0.004 | SO_4_^2-^ | 0.078 | Al | 0.38 | SO_4_^2-^ | 0.057 |

**Table S2.** Elemental concentrations of the leachates before precipitation

| **Element** | **Commercial-LFP leachate (FP-B), g L^-1^** | **Spent-LFP leachate (FP-C), g L^-1^** |
| --- | --- | --- |
| Li | 1.379 | 0.883 |
| Fe | 11.990 | 8.470 |
| P | 6.466 | 3.657 |
| Ca | 0.0317 | 0.0643 |
| Mg | 0.0037 | 0.0063 |
| Na | 0.0180 | 0.0267 |
| K | 0.0190 | 0.0240 |
| Si | 0.0057 | 0.0447 |
| Al | 0.0013 | 0.1200 |
| Mn | 0.0017 | 0.0993 |
| Zn | 0.0010 | 0.0020 |
| Pb | 0.0003 | 0.0003 |
| Cu | 0 | 0.0470 |
| B | 0.0067 | 0.0057 |
| Ni | 0 | 0.2407 |
| Cathode-derived sulfate equivalent | 0.0233 | 0.0157 |
| Molar P/Fe ratio before phosphate adjustment | 0.972 | 0.778 |
| Note. Elemental concentrations were measured in the leachates before precipitation. The molar P/Fe ratios were calculated from the measured Fe and P concentrations. | | |

**Table S3.** Lithium distribution during leaching and FePO_4_ regeneration.

| **Process stream** | **FP-B** | | **FP-C** | |
| --- | --- | --- | --- | --- |
|  | **Li amount**  **(mg)** | **Fraction of**  **feed Li (%)** | **Li amount**  **(mg)** | **Fraction of**  **feed Li (%)** |
| Initial Li in the LFP feed | 42.000 | 100.000 | 27.000 | 100.000 |
| Li in the leachate (intermediate stream) | 41.370 | 98.500 | 26.490 | 98.111 |
| Li loss during leaching | 0.630 | 1.500 | 0.510 | 1.889 |
| Li in the primary filtrate | 40.713 | 96.936 | 26.192 | 97.008 |
| Li in washing liquors (cycles 1-3) | 0.467 | 1.111 | 0.237 | 0.878 |
| Li retained in the washed precipitate | 0.190 | 0.453 | 0.061 | 0.226 |
| Li associated with the unwashed precipitate | 0.657 | 1.564 | 0.298 | 1.103 |
| Note. The data in this table and Figure 2c were obtained from different experimental batches. The differences in the reported proportions reflect batch-to-batch variation. | | | | |

**Table S4.** Sequential Li release during washing

| **Washing cycle** | **FP-B (%)** | **FP-C (%)** |
| --- | --- | --- |
| 1 | 0.581730 | 0.488889 |
| 2 | 0.360360 | 0.322222 |
| 3 | 0.185970 | 0.083333 |
| 4 | 0.006330 | 0.012444 |
| 5 | 0.000892 | 0.011556 |
| Total, cycles 1-3 | 1.128060 | 0.894444 |
| Total, cycles 1-5 | 1.135282 | 0.918444 |

**Table S5.** Fe–P mass balance for FP-B and FP-C.

| **Process stream** | **FP-B Fe (mg)** | **FP-B P (mg)** | **FP-C Fe (mg)** | **FP-C P (mg)** |
| --- | --- | --- | --- | --- |
| Initial leachate | 359.700 | 193.980 | 254.100 | 109.710 |
| Additional input from Na_3_PO_4_·12H_2_O | 0 | 0 | 0 | 27.297 |
| Washing liquors | 0.048900 | 0.765 | 0.003510 | 0.630 |
| Primary filtrate after precipitation | 4.000 | 10.099 | 10.976 | 1.700 |
| Washed precipitate | 355.652 | 183.116 | 243.120 | 134.677 |

**Table S6.** Experimental conditions for the single-factor precipitation experiments.

| **Varied parameter** | **Investigated range** | **Fixed pH** | **Fixed temperature (°C)** | **Fixed reaction time (min)** | **Fixed H_2_O_2_/leachate volume ratio** |
| --- | --- | --- | --- | --- | --- |
| **pH** | 1.0-3.0 | — | 35 | 60 | 1:60 |
| **Temperature** | 35-95 °C | 2.0 | — | 60 | 1:60 |
| **Reaction time** | 10-90 min | 2.0 | 80 | — | 1:60 |
| **H_2_O_2_ dosage** | 1:110-1:10 | 2.0 | 80 | 50 | — |

**Table S7**. Kinetic fitting parameters for FP-B and FP-C.

| **Sample** | **Temperature (°C)** | **ln k ± SE** | **k (min^-n^)** | **n ± SE** | **R^2^** |
| --- | --- | --- | --- | --- | --- |
| **FP-B** | 35 | −1.67844 ± 0.19562 | 0.1867 | 0.51849 ± 0.05211 | 0.97059 |
|  | 50 | −1.44904 ± 0.13825 | 0.2348 | 0.50750 ± 0.03683 | 0.98445 |
|  | 65 | −1.06280 ± 0.18428 | 0.3455 | 0.48902 ± 0.04909 | 0.97066 |
|  | 80 | −0.93886 ± 0.18389 | 0.3911 | 0.52481 ± 0.04898 | 0.97453 |
|  | 95 | −0.78644 ± 0.28251 | 0.4555 | 0.51796 ± 0.07525 | 0.94045 |
| **FP-C** | 35 | −1.72124 ± 0.17299 | 0.1788 | 0.48307 ± 0.04608 | 0.97343 |
|  | 50 | −1.34244 ± 0.18336 | 0.2612 | 0.47122 ± 0.04884 | 0.96878 |
|  | 65 | −0.91713 ± 0.20429 | 0.3997 | 0.46300 ± 0.05442 | 0.96020 |
|  | 80 | −0.65179 ± 0.09525 | 0.5211 | 0.45912 ± 0.02537 | 0.99092 |
|  | 95 | −0.55248 ± 0.11074 | 0.5755 | 0.46345 ± 0.02950 | 0.98799 |
| **Sample** | **Intercept a ± SE** | **Slope b ± SE** | **R^2^** | **Formal Ea (kJ mol^-1^)** | |
| **FP-B** | 3.99864 ± 0.49134 | −1.74452 ± 0.16509 | 0.97384 | +14.50 ± 1.37 | |
| **FP-C** | 5.81927 ± 0.65488 | −2.30827 ± 0.22004 | 0.97346 | +19.19 ± 1.83 | |

**Table S8.** Input compositions and diagnostic parameters for the conditional hot-filtrate models.

| **Sample** | **Temperature(°C)** | **Hot-filtrate pH** | **Total Li (mol L^-1^)** | **Total Fe (III) (mol L^-1^)** | **Total P (mol L^-1^)** | **I (mol kg^-1^)** | **CBE (%)** |
| --- | --- | --- | --- | --- | --- | --- | --- |
| **FP-B** | 35 | 1.90 | 0.149925 | 0.041798 | 0.035003 | 4.88 | -10.81 |
|  | 50 | 1.80 | 0.149833 | 0.021333 | 0.019355 | 4.67 | -8.96 |
|  | 65 | 1.65 | 0.149271 | 0.009958 | 0.008318 | 4.32 | -5.32 |
|  | 80 | 1.50 | 0.149773 | 0.001827 | 0.008318 | 3.91 | -0.59 |
|  | 95 | 1.00 | 0.149681 | 0.000777 | 0.000651 | 3.06 | +13.39 |
| **FP-C** | 35 | 1.85 | 0.096348 | 0.030922 | 0.033027 | 4.88 | -10.11 |
|  | 50 | 1.71 | 0.096250 | 0.016262 | 0.017543 | 4.61 | -7.39 |
|  | 65 | 1.64 | 0.096192 | 0.012373 | 0.009142 | 4.35 | -4.51 |
|  | 80 | 1.53 | 0.096123 | 0.005014 | 0.001400 | 3.99 | -0.52 |
|  | 95 | 1.41 | 0.096114 | 0.002089 | 0.000700 | 3.63 | +4.18 |
| **Note.** The listed concentrations are PHREEQC inputs, not measured activities. I, calculated ionic strength; CBE, charge-balance error. The estimated total Na concentrations were 2.112844 and 2.180580 mol L^-1^ for FP-B and FP-C, respectively; the corresponding estimated total S(VI) concentrations were 1.530798 and 1.530612 mol L^-1^. The FP-C input includes 0.335 g Na₃PO₄·12H_2_O per experiment. Trace-element inputs were based on feed-composition estimates rather than separate measurements of every final filtrate. | | | | | | | |

**Table S9.** Calculated free-ion activities in the SIT model.

| **Sample** | **Temperature (°C)** | **a(Li^+^)** | **a(Fe^3+^)** | **a(H_2_PO_4_^-^)** | **a(PO_4_^3-^)** | **a(SO_4_^2-^)** |
| --- | --- | --- | --- | --- | --- | --- |
| **FP-B** | 35 | 0.102 | 1.07×10^-6^ | 1.50×10^-3^ | 3.31×10^-19^ | 0.0692 |
|  | 50 | 0.0998 | 7.93×10^-7^ | 1.23×10^-3^ | 2.39×10^-19^ | 0.0599 |
|  | 65 | 0.0979 | 6.53×10^-7^ | 6.46×10^-4^ | 8.46×10^-20^ | 0.0479 |
|  | 80 | 0.0968 | 1.48×10^-7^ | 7.33×10^-4^ | 6.34×10^-20^ | 0.0354 |
|  | 95 | 0.0965 | 2.12×10^-7^ | 2.42×10^-5^ | 2.69×10^-22^ | 0.0138 |
| **FP-C** | 35 | 0.0654 | 7.54×10^-7^ | 2.03×10^-3^ | 3.56×10^-19^ | 0.0669 |
|  | 50 | 0.0643 | 7.19×10^-7^ | 1.32×10^-3^ | 1.68×10^-19^ | 0.0563 |
|  | 65 | 0.0632 | 8.50×10^-7^ | 6.39×10^-4^ | 7.99×10^-20^ | 0.0465 |
|  | 80 | 0.0622 | 5.46×10^-7^ | 1.06×10^-4^ | 1.05×10^-20^ | 0.0356 |
|  | 95 | 0.0611 | 2.78×10^-7^ | 4.87×10^-5^ | 3.58×10^-21^ | 0.0254 |
| **Note.** Activities are dimensionless. a(Li^+^) denotes the activity of free aqueous Li^+^, not the total dissolved Li concentration. | | | | | | |

**Table S10.** Calculated Fe–sulfate complex fractions and saturation indices of the hot filtrates.

| **Sample** | **Temperature (°C)** | **FeSO_4_^+^/total Fe (III) (%)** | **Fe(SO_4_)_2_^-^/total Fe (III) (%)** | **SI: FePO_4_(cr)** | **SI: FePO_4_·2H_2_O** |
| --- | --- | --- | --- | --- | --- |
| **FP-B** | 35 | 6.53 | 30.00 | -3.121 | +0.418 / -0.612 / +1.959 |
|  | 50 | 13.38 | 33.24 | -2.866 | +0.296 / -0.671 / +1.760 |
|  | 65 | 29.46 | 38.12 | -2.921 | -0.094 / -0.985 / +1.289 |
|  | 80 | 40.44 | 26.09 | -3.252 | -0.722 / -1.523 / +0.577 |
|  | 95 | 75.98 | 13.29 | -5.066 | -2.807 / -3.501 / -1.594 |
| **FP-C** | 35 | 6.00 | 26.64 | -3.242 | +0.297 / -0.733 / +1.838 |
|  | 50 | 14.93 | 34.88 | -3.060 | +0.101 / -0.865 / +1.565 |
|  | 65 | 29.85 | 37.43 | -2.831 | -0.004 / -0.895 / +1.379 |
|  | 80 | 54.61 | 35.43 | -3.467 | -0.936 / -1.738 / +0.362 |
|  | 95 | 69.42 | 22.38 | -3.823 | -1.557 / -2.251 / -0.344 |
| **Note.** Complex fractions were calculated from species molalities relative to total dissolved Fe (III), not from activities. The three FePO_4_·2H_2_O SI values in each cell are ordered Thermoddem / LLNL / MINTEQ. They represent a sensitivity test of solid-phase reference constants applied to the same SIT aqueous-speciation result, not three independent aqueous models. All indices describe post-precipitation hot filtrates. | | | | | |

**Table S11.** Database sensitivity of aqueous Li–sulfate association.

| **Sample** | **Temperature (°C)** | **WATEQ4F** | **MINTEQ v4** | **LLNL** |
| --- | --- | --- | --- | --- |
| **FP-B** | 35 | 0.0897 / 0.0257 / 23.51% | 0.1015 / 0.0532 / 47.16% | 0.1015 / 0.0472 / 35.38% |
|  | 50 | 0.0911 / 0.0231 / 21.40% | 0.1106 / 0.0460 / 41.33% | 0.1048 / 0.0429 / 32.59% |
|  | 65 | 0.0935 / 0.0192 / 18.10% | 0.1246 / 0.0369 / 33.77% | 0.1104 / 0.0357 / 27.70% |
|  | 80 | 0.0976 / 0.0146 / 13.88% | 0.1416 / 0.0279 / 25.94% | 0.1185 / 0.0265 / 21.04% |
|  | 95 | 0.1057 / 0.0058 / 5.54% | 0.1813 / 0.0121 / 11.50% | 0.1344 / 0.0098 / 8.00% |
| **FP-C** | 35 | 0.0580 / 0.0163 / 23.15% | 0.0667 / 0.0331 / 45.58% | 0.0657 / 0.0299 / 34.87% |
|  | 50 | 0.0593 / 0.0143 / 20.59% | 0.0743 / 0.0279 / 38.90% | 0.0685 / 0.0265 / 31.42% |
|  | 65 | 0.0607 / 0.0122 / 17.78% | 0.0817 / 0.0235 / 33.30% | 0.0716 / 0.0228 / 27.36% |
|  | 80 | 0.0626 / 0.0095 / 14.06% | 0.0908 / 0.0184 / 26.59% | 0.0759 / 0.0175 / 21.49% |
|  | 95 | 0.0647 / 0.0066 / 9.94% | 0.1009 / 0.0137 / 20.17% | 0.0808 / 0.0119 / 14.90% |
| **Note.** Each database entry is ordered a(Li^+^) / a(LiSO_4_^-^) / LiSO_4_^-^ as a percentage of total dissolved Li. Activities are dimensionless. Each database was run as a complete, independent aqueous model using the same solution inputs; its activities should not be combined numerically with the SIT results in Table S9. LiSO_4_^-^ is an aqueous ion pair, not a solid phase. | | | | |
